# Helminths-based bi-functional molecule, tuftsin-phosphorylcholine (TPC), ameliorates an established murine arthritis

**DOI:** 10.1371/journal.pone.0200615

**Published:** 2018-08-08

**Authors:** Miri Blank, Tomer Bashi, Jordan Lachnish, Dana Ben-Ami-Shor, Ora Shovman, Mati Fridkin, Miriam Eisenstein, Alexander Volkov, Iris Barshack, Yehuda Shoenfeld

**Affiliations:** 1 Zabludowicz Center for Autoimmune Diseases, Sheba Medical Center, affiliated with Sackler Faculty of Medicine, Tel Aviv University, Tel Aviv, Israel; 2 Department of Organic Chemistry, Weizmann Institute of Science, Rehovot, Israel; 3 Department of Chemical Research Support, Weizmann Institute of Science, Rehovot, Israel; 4 Institute of Pathology, Sheba Medical Center, affiliated with Sackler Faculty of Medicine, Tel Aviv University, Tel Aviv, Israel; Universite de Nantes, FRANCE

## Abstract

A novel small molecule named tuftsin-phosphorylcholine (TPC), which is linked to the biological activity of helminths, was constructed. The current study address the effect of TPC treatment in established collagen-induced arthritis (CIA) mice and propose TPC bi-functional activity. TPC treatment was initiated when clinical score was 2 to 4. Arthritis scores in TPC treated mice were lower compared to mice treated with vehicle (*P* < 0.001). Joint staining showed normal joint structure in TPC-treated mice compared to control groups treated with phosphate buffered saline (PBS), phosphorylcholine, or tuftsin, which exhibited severely inflamed joints. TPC enhanced anti-inflammatory response due to increased IL-10 secretion, and reduced pro-inflammatory cytokine secretion (IL-1-β, IL-6, TNF-α*P* < 0.001). Furthermore, TPC therapy increased expansion of CD4^+^CD25^+^FOXP3^+^T regulatory cells and IL-10^+^CD5^+^CD1d^+^B regulatory cells. We propose that the immunomodulatory activity of TPC can be a result of a bi-specific activity of TPC: (a) The tuftsin part of the TPC shifts RAW macrophage cells from pro-inflammatory macrophages M1 to anti-inflammatory M2-secreting IL-10 (*P* < 0.001) through neuropilin-1 and (b) TPC significantly reduce mouse TLR4 expression *via* NFkB pathway by HEK^TM^ cells (*P* < 0.02) *via* the phosphorylcholine site of the molecule. Our results indicate that TPC, significantly ameliorated established CIA by its immunomodulatory activity. These data could lead to a novel self bi-functional small molecule for treating patients with progressive RA.

## Introduction

Rheumatoid arthritis (RA) is a chronic, systemic autoinflammatory disease. It usually manifests as stiffness, pain, and swelling of the joints [1]. Similar to other autoimmune diseases, RA has a genetic background associated mainly with HLA-DRB1, PTPN22, and TRAF1-C5 [2,3]. Environmental factors also contribute to the development of RA; the most prominent of these are smoking and infections, such as Epstein-Barr virus (EBV) infection [4,5]. One of the main explanations for the joint inflammation is the presence of citrullinated proteins followed by production of antibodies targeting these proteins (anti-citrullinated protein antibodies [ACPAs]) [6]. In RA patients, the function of T regulatory (Treg) cells is impaired and increases Treg cellscount is well-correlated with a better clinical response in patients and animal models [7,8]. Furthermore, several studies of cells derived from RA patients indicated that the number of B10 regulatory (Breg) cells was inversely correlated with RA severity [9]. Impaired Breg activity and high levels of IFN-γ expressing cells suppressed Treg cell differentiation and worsened arthritis in a murine model [10].

The use of disease-modifying agents such methotrexate (traditionally used in RA treatment as a classic first choice disease modifying antirheumatic drug) and biologic agents such as anti-TNF-α, anti-IL-1, anti-IL-6, and anti-CD20 blockers was associated with various side effects, including increased rate of infections [11–15]. Therefore, new small immunomodulatory molecules with minimal side effects are needed for treating patients. Adopting a natural -based strategy to immunomodulate the host immune network could be a beneficial approach for treatment.

The burden of infections has decreased in the industrial world [16]. Yet, inverse correlation between the prevalence of helminthes infections in endemic areas and RA was observed [17]. Helminths survive within the host by adapting the host immune network for their benefit. The clinical activity score of RA improved in patients and animals treated with live helminths or helminth products [18–20]. The immunoregulatory functions of some helminths were shown to be a results of phosphorylcholine moiety on the helminths’ secretory molecules [21]. Phosphorylcholine, like other phospholipids, is non-immunogenic. Thus, we conjugated phosphorylcholine to tuftsin (a self-immunomodulatory molecule produced by the spleen) [22] and created a novel chimeric molecule–tuftsin-phosphorylcholine, coined TPC. Previously, we have demonstrated that preventive treatment with TPC inhibited glomerulonephritis in mice genetically prone to lupus. Moreover, TPC prevented severity of colitis in dextran sulfate sodium (DSS) salt-induced murine colitis. Finally, TPC attenuated the development of joint destruction and arthritis score in murine collagen-induced arthritis (CIA) [23–25]. The current study assesses the therapeutic efficacy of TPC in established murine arthritis and proposes a mechanism for TPC immunomodulatory activity.

## Materials and methods

### Synthesis of Tuftsin-phosphorylcholine (TPC)

Tuftsin (GLS peptide synthesis, Shanghai, China) was coupled to diazotized 4-aminophenyphosphorylchloride (Biosearch Technologies, Inc. Novato, CA, USA) to form an azo bond between the tuftsin and phosphorylcholine, named TPC, by Prof. Mati Fridkin (Department of Organic Chemistry at Weizmann Institute of Science, Rehovot, Israel). The conjugate was characterized by mass spectra, amino acid analysis, as well as by high-performance liquid chromatography (HPLC). TPC was diluted in phosphate buffered saline (PBS) (Biological Industries Israel Beit-Haemek Ltd, Kibbutz Beit-Haemek, Israel).

### Mice and experimental design

Experimental arthritis was induced in 6–7 week old DBA/1J male mice (ENVIGO, Blackthorn, UK). The mice were maintained in a conventional animal housing facility at Sheba Medical Center and kept in individually ventilated cages. All experiments were approved and executed according to the protocols of the ethics committee of the Israeli Ministry of Health (no.696/11 and 1008/16). Collagen induced arthritis (CIA) was performed as follow: Bovine type II collagen (Chondrex, Redmond, WA, USA) was emulsified 1:1 with mycobacterium tuberculosis H37RA in Freund’s incomplete adjuvant (Difco Laboratories, Detroit, MI, USA). DBA/1J males were subcutaneously injected into the base of the tail with 100 μg emulsion. A boost injection of bovine type II collagen in PBS, at the base of the tail, was given 16 days later. TPC therapy started at score of 2–4, 5 μg/0.1 ml/mouse 3 time per week. Comparative groups were given PBS, tuftsin, or phosphorylcholine at the same protocol as TPC, n = 15 per each group. CIA untreated mice group was used as a control. The mice were sacrificed after 35 days.

### Assessment of arthritis

Mice were monitored twice weekly by two blind observers for signs of arthritis. The severity of disease scores were defined as follows: 0 = normal, 1 = slight erythema, 2 = slight erythema plus swelling, 3 = moderate edema and erythema, 4 = edema and erythema from the ankle to the entire leg. The total arthritis score was the sum score of the four limbs. The paws of the mice were obtained from the sacrificed mice and fixed in 4% formalin (Sigma-Aldrich St Louis, MO, USA), decalcified, cut, and stained with H&E. All histological evaluations performed by pathologists were double blinded.

### *In-vitro* analysis of cytokine production by splenocytes derived from the studied mice

Spleen cells were derived from TPC treated mice, as well as from mice treated with tuftsin, phosphorylcholine or vehicle PBS, (n = 10 mice per group). Red blood cells were lysed by using red-blood-cells lysis buffer (Biological Industries Israel Beit-Haemek Ltd). The spleen cells were seeded (5 × 10^5^ cells/well) in 24-well plates (Nunclon^TM^, Roskilde, Denmark), precoated with anti-CD3 Abs (2 μg/ml). TPC, tuftsin, or phosphorylcholine at concentration of 5 μg/ml, or PBS (vehicle) were added to the cultures, in RPMI1640 enriched medium supplemented with 10% heat-inactivated fetal bovine serum, 2 mM L-glutamine, 1 mM sodium pyruvate, 0.1 mM non-essential amino acids, 100 U/ml penicillin, 100 μg/ml streptomycin, and 50 μM 2-mercaptoethanol (Biological Industries Beit-Haemek Ltd, Israel). After 72hrs of incubation at 37°C and 5% CO_2,_ the supernatants were collected. Pro-inflammatory (IL-1β, IL-17, IL-6, TNF-α) and IL-10 anti-inflammatory cytokine levels in the culture supernatant were detected by DuoSet ELISA kits (R&D systems Minneapolis, MN, USA) according to manufacturer instructions.

### Analysis of T regulatory (CD4^+^CD25^+^FOXP3^+^) cells and B10 regulatory (IL-10^+^ CD1d^+^CD5^+^) cells by flow cytometry

Isolated splenocytes were depleted of red blood cells. The cells were incubated with anti-CD4^+^FITC anti-CD25^+^APC anti-FOXP3^+^PE (eBioscience, San Diego, CA, USA) for Tregs and anti-mouse CD304 (Neuropilin-1) PerCP-eFluor®710 (eBioscience) for specific neuropilin-1 positive Tregs. B cells isolated in splenocytes undergo negative selection using monoclonal antibodies against CD43, CD4, and Ter-119 (B cell isolation kit—Miltenyi Biotec, Auburn, CA, USA). B cells were incubated with anti-IL-10^+^FITC anti-CD1d^+^APC anti-CD5^+^PE (eBioscience) and analyzed by flow cytometry with forward and side scatter gates adjusted to include all cells and to exclude debris (Becton Dickinson, Franklin Lakes, NY, USA). For intracellular staining of FOXP3, the cells were pre-incubated with a fixation solution, washed, and resuspended in permeabilization solution (Serotec, Oxford, UK) and intracellularly stained for FOXP3. The gating for Tregs was on the CD4^+^ T cells and the gating for Bregs was on IL-10^+^ B cells. The flow cytometry used was from Becton Dickinson, Franklin Lakes, NY, USA.

### *In-vitro* shift of macrophages from M1 toward M2 by TPC

Murine macrophage RAW 264.7 cell line (Sigma-Aldrich) were cultured in RPMI1640 with 10% fetal calf serum, penicillin, and streptomycin (all from Biological Industries Israel Beit-Haemek Ltd) in 24-well tissue culture plates,1 × 10^6^ cells/well. For inducing differentiation from M0 to M1 macrophages, secreting pro-inflammatory cytokines IL-6 and TNF-α, the cells were incubated with 10 ng/ml LPS (Sigma-Aldrich) and 20 ng/ml recombinant mouse IFN-γ (R&D systems Minneapolis, MN, USA) for 24 hours. Differentiation from M1 to M2 macrophages secreting anti-inflammatory cytokine IL-10, the M1cells were exposed to 20 ng/ml recombinant mouse IL-4 (R&D systems Minneapolis, MN, USA) for 48 hours. In order to show the ability of TPC, tuftsin or phosporylcholine to shift M1 to M2 cells, the compounds at 5 μg/ml were added to M1 cells for 48 hours. Culture supernatants were then collected. Pro-inflammatory IL-6, TNF-α cytokines, and IL-10 anti-inflammatory cytokine levels in the culture supernatant were measured by DuoSet ELISA kits (R&D systems Minneapolis, MN, USA) according to the manufacturer instructions. Based on previous studies showing the neuropilin-1 as target molecule for tuftsin on brain microglioma cells, we tested the TPC effect on the neuropilin-1 [26]. To define the neuropilin-1 on macrophages through which TPC may affect the M1 phenotype switch, we incubated the cells with neuropilin-1 inhibitor, 50μM EG00229 N^2^-[[3-[(2,1,3-Benzothiadiazol-4-ylsulfonyl)amino]-2-thienyl]carbonyl]-L-arginine (Tocris Bioscience, Ellisville, MO, USA) for 1 hour before adding the TPC, tuftsin or phosphorylcholine.

### Neuropilin-1 (NRP1) expression by M2 macrophages, using RT-PCR

Neuropilin-1 mRNA expression by RT-PCR was conducted as previously reported [27]. Total mRNA was isolated from M2 RAW cells treated with TPC or a vehicle, using the RNeasy Mini Kit (Qiagen, Hilden, Germany), followed by RT-PCR reaction. Isolated total mRNA was reverse-transcribed to prepare cDNA by using Maloney murine leukemia virus reverse transcriptase (Promega Inc., Madison, WI, USA). The resulting cDNA was subjected to RT- PCR in the presence of specific primers. The following primer pairs were used: mouse NRP-1 a domain (404bp) F5′-GGC TGC CGT TGC TGT GCG-3′ and R5′-ATA GCG GAT GGA AAA CCC-3′; GAPDH F5′-ACC CCT TCA TTG ACC TCA ACT-3′ and R5′-CCACCA CCCTGTTGCTGTAG-3′. Briefly, a 20 μl reaction volume contained 3 mM MgCl_2_, Light Cycler HotStart DNA SYBR Green I mix (Roche, Basel, Switzerland), specific primer pairs, and 5 μl of cDNA. The levels of GAPDH were used to normalize the gene expression levels of neuropilin-1.

### Molecular modeling of TPC

A model of the binding of tuftsin to neuropilin was constructed base on the X-ray structure of neuropilin in complex with a short peptide KPR which consists as part of tuftsin (TKPRGY) [28]. Tuftsin residue was added at the N-terminus of KPR. Tuftsin linker residues, GY, were added at the C-terminus of KPR by forming peptide bond with either of the terminal C = O groups. The resultant two models were subjected to energy minimization and 50 ns molecular dynamics (MD) of the neuropilin/tuftsin complex immersed in water and neutralized. In one of the complexes the peptide moved out of the binding site, whereas in the other complex the peptide remained bound and the structural changes in neuropilin were minor. This model was used to manually attach phosphocholine to the exposed OH group of the C-terminal tyrosine, followed with short energy minimization. Computations were performed with Gromacs [29]. Visualization of the molecules, energy minimization of the tuftsin-phosphocholin chimera, was conducted with UCSF-Chimera (Confirm) [30].

### HEK-blueTM-TLR4 cell culture signaling assay

We used human embryonic kidney (HEK239)-Blue- mTLR4 cells (cat.no # hkb-mtlr4 cells, InvivoGen, San Diego, CA, USA) according to manufacturer protocol. The system comprises of: HEK239-Blue- mTLR4 cells that were obtained by co-transfection of the murine TLR4, MD-2, and CD14 (part of LPS signaling resulting in NF-κB translocation) co-receptor genes. In co-transfection of HEK/mTLR4 cells with pNiFty- SEAP (secreted embryonic alkaline phosphatase) reporter gene and Pduo2-Mmd2/CD14 plasmids. We used the transfection reagent LyoVecTM (InvivoGen). The SEAP reporter gene was placed under the control of an IL-12 p40 minimal promoter fused to five NF-κB and AP-1 binding sites. Stimulation with a LPS (TLR4 agonist) activated NF-κB and AP-1 which induced the production of SEAP assessed by a colorimetric assay. HEK-Blue™ mTLR4 cells were stimulated for 24 hours with agonist LPS-EB (InvivoGen) ultrapure (10 ng/ml). TPC, tuftsin, or phosphorylcholine (1 μg/ml, 10 μg/ml, 25 μg/ml, 50 μg/ml, 100 μg/ml) were added before stimulation with LPS. In several cases the inhibitor for LPS [1-palmitoyl-2-arachidonyl-snglycero-3-phosphorylcholine (OxPAPC)] at 30 μg/ml was added for 1 hour before TPC, tuftsin, or phosphorylcholine and were applied for 24 hours. Later, we applied cell culture supernatant to QUANTI-Blue medium (InvivoGen) and measured alkaline phosphatase activity at 655 nm OD after approximately 30 minutes. The level of SEAP released into the culture media was used to quantify the extent of TLR4 stimulation/inhibition.

### Statistical analysis

Differences among the studied groups were tested using analysis of variance (ANOVA) according to the data. The analyses that were statistically significant were followed by Tukey's test to identify specific differences between groups. Statistical analysis was performed using SPSS software version 17 (SPSS Inc., Chicago, IL, USA) Results are shown as averages ± standard deviation. Values of *P* < 0.05 were considered significant; *P* reported values correspond to Tukey's test for specific group differences.

## Results

### TPC inhibited arthritis progression in CIA mice

**Fig 1:**
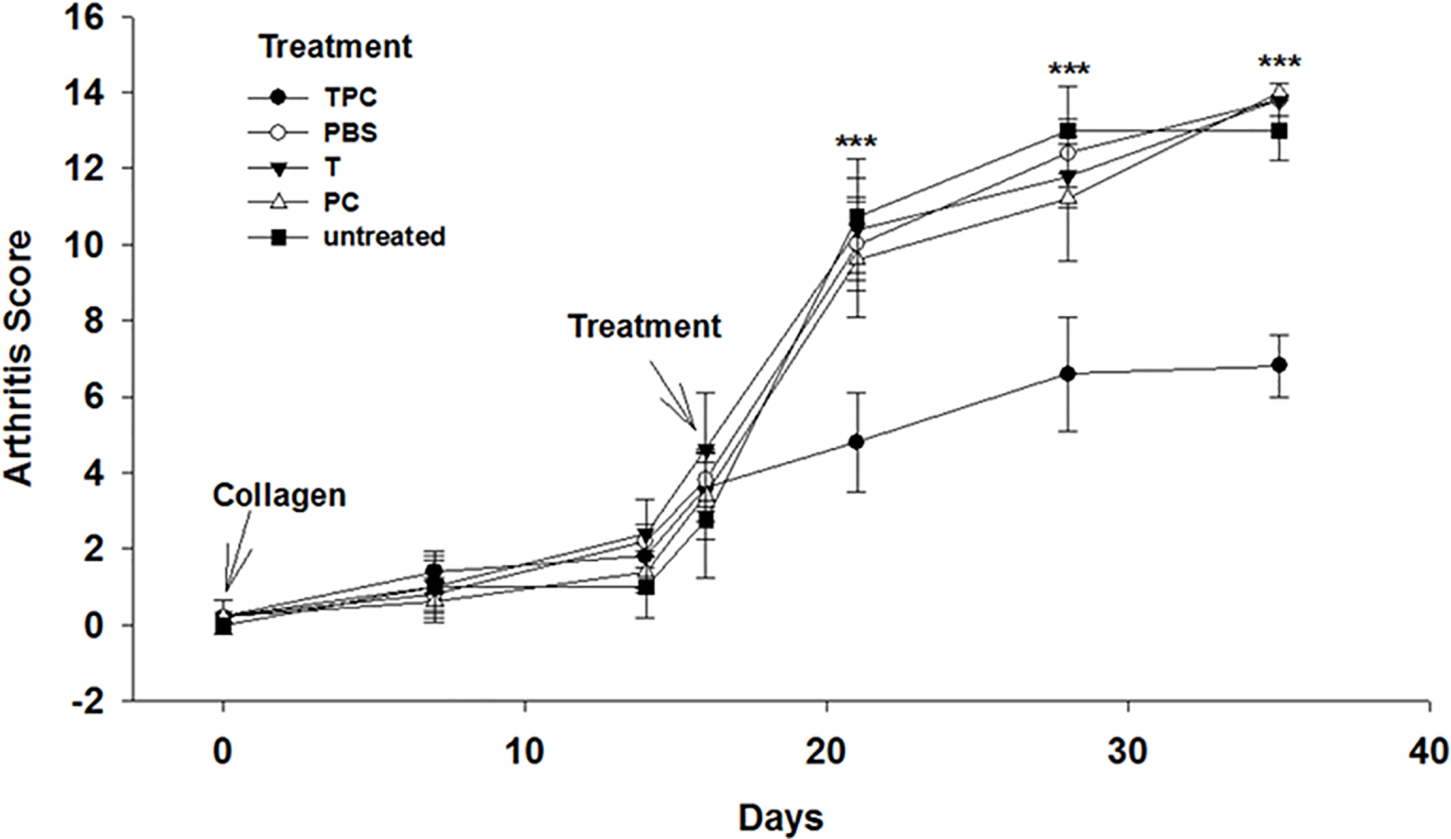
The effect of TPC on arthritis scores in DBA/1J CIA mice. The data are presented as arthritis score of s.c. treated CIA mice, measured from day (0) (disease induction) until day 35 mean ± SD. Tuftsin-phosphorylcholine (TPC) (n = 10), PBS (n = 10), tuftsin (T) (n = 10), phosphorylcholine (PC) (n = 10), untreated (n = 10). Values are mean ± SD. ***P < 0.001.

**Fig 2:**
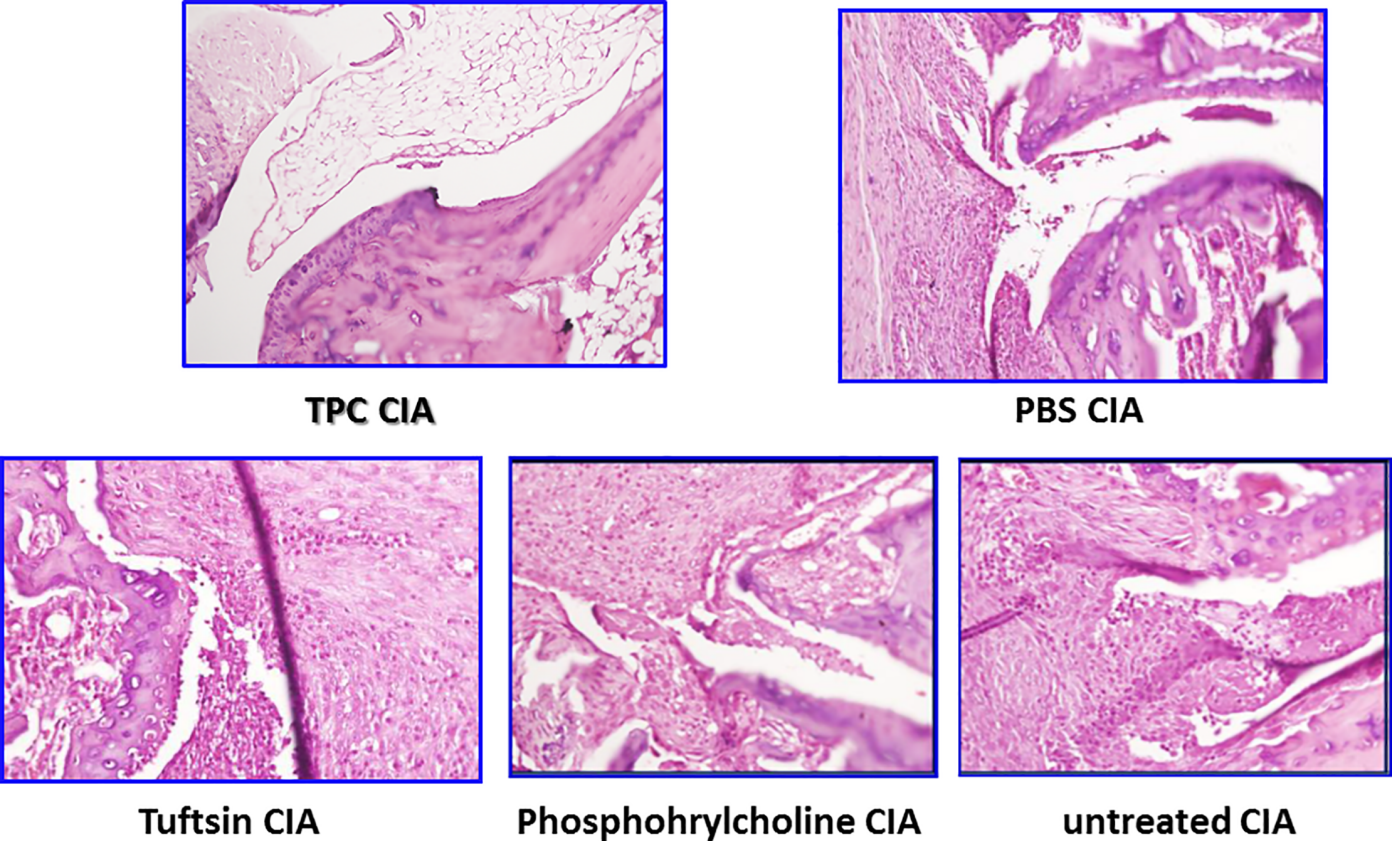
Histological analysis. Representative arthritic paws from each study group of CIA mice were removed and stained with H&E. Magnification presented × 100.

CIA is a mouse model which imitates rheumatoid arthritis in genetically prone human patients, in which inflammation leads to the joint destruction. We investigated the effect of TPC administration in CIA mice and compared it with CIA mice treated, following disease establishment, with tuftsin (T), phosphorylcholine (PC), or PBS. The treatment started when the baseline score was defined as follows by groups: TPC 3.6 ± 0.9, PBS 3.8 ± 0.8, tuftsin 4.6 ± 1.5, phosphorylcholine 3.4 ± 1.1, and untreated 2.75 ± 1.5, *P* > 0.05) (Fig 1). Seven days after exposure to TPC, we observed a significantly lower arthritis score in TPC-treated mice compared to control CIA mice (*P* < 0.001). The significantly lower arthritis score lasted until the mice were sacrificed at day 35 (scores: TPC 6.8 ± 0.8, PBS 13.8 ± 0.45, tuftsin 13.1 ± 0.64, phosphorylcholine 14±0.4 and untreated 12 ± 0.8, *P* < 0.001) (Fig 1, S9 File). H&E staining of joints sections from the TPC-treated mice, demonstrated significantly less synovial hyperplasia, normal cartilage layer and muscle structure, typical bone organization and uninflamed fat tissue (Fig 2). H&E staining of the joints from PBS, tuftsin, and phosphorylcholine treated CIA mice exhibited high levels of inflammation, large areas of fibrosis, and several spots of necrosis, compared to TPC treated mice. Likewise, severe bone and muscles destruction was seen in these comparative groups of mice (Fig 2).

### TPC treatment of mice with CIA, regulated the cytokines secretion *in-vitro* by splenocytes

**Fig 3:**
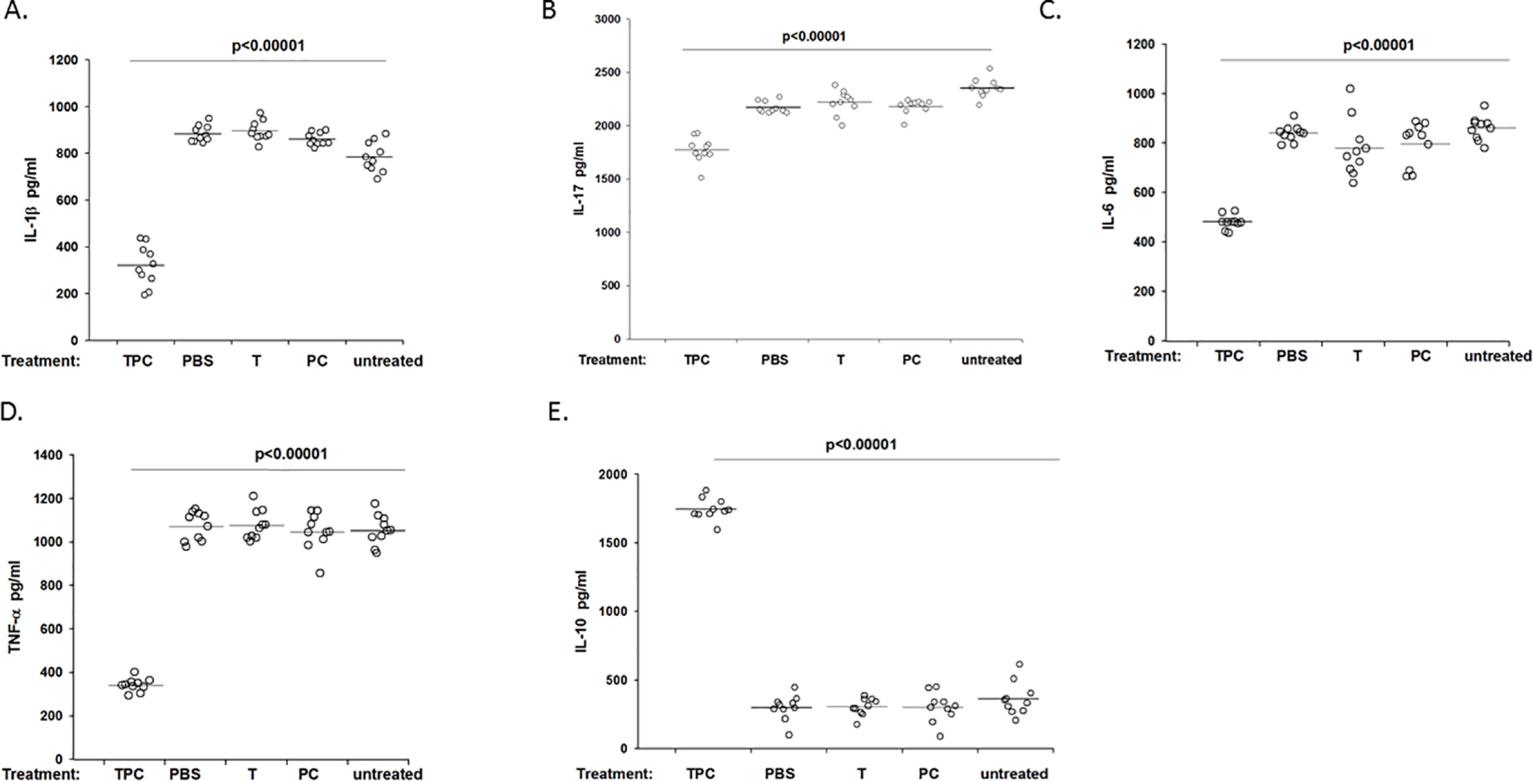
Cytokine expression by splenocytes from CIA mice. In vitro analyses of **concentrations** of the pro-inflammatory cytokines IL-1β, IL-17, IL-6, TNF-α, and the anti-inflammatory cytokine IL-10, in the culture fluids of splenocytes originating from tuftsin-phosphoryl choline (TPC), PBS, tuftsin (T), phosphorylcholine (PC) and untreated CIA mice. The data are presented as concentration in pg/ml. n = 10 per group. [A] In vitro analyses of the pro-inflammatory cytokine IL-1-β concentration. [B] In vitro analyses of the pro-inflammatory cytokine IL-17 concentration. [C] In vitro analyses of the pro-inflammatory cytokine IL-6 concentration. [D] In vitro analyses of the pro-inflammatory cytokine TNF-α concentration. [E] In vitro analyses of the anti-inflammatory cytokine IL-10 concentration.

**Fig 4:**
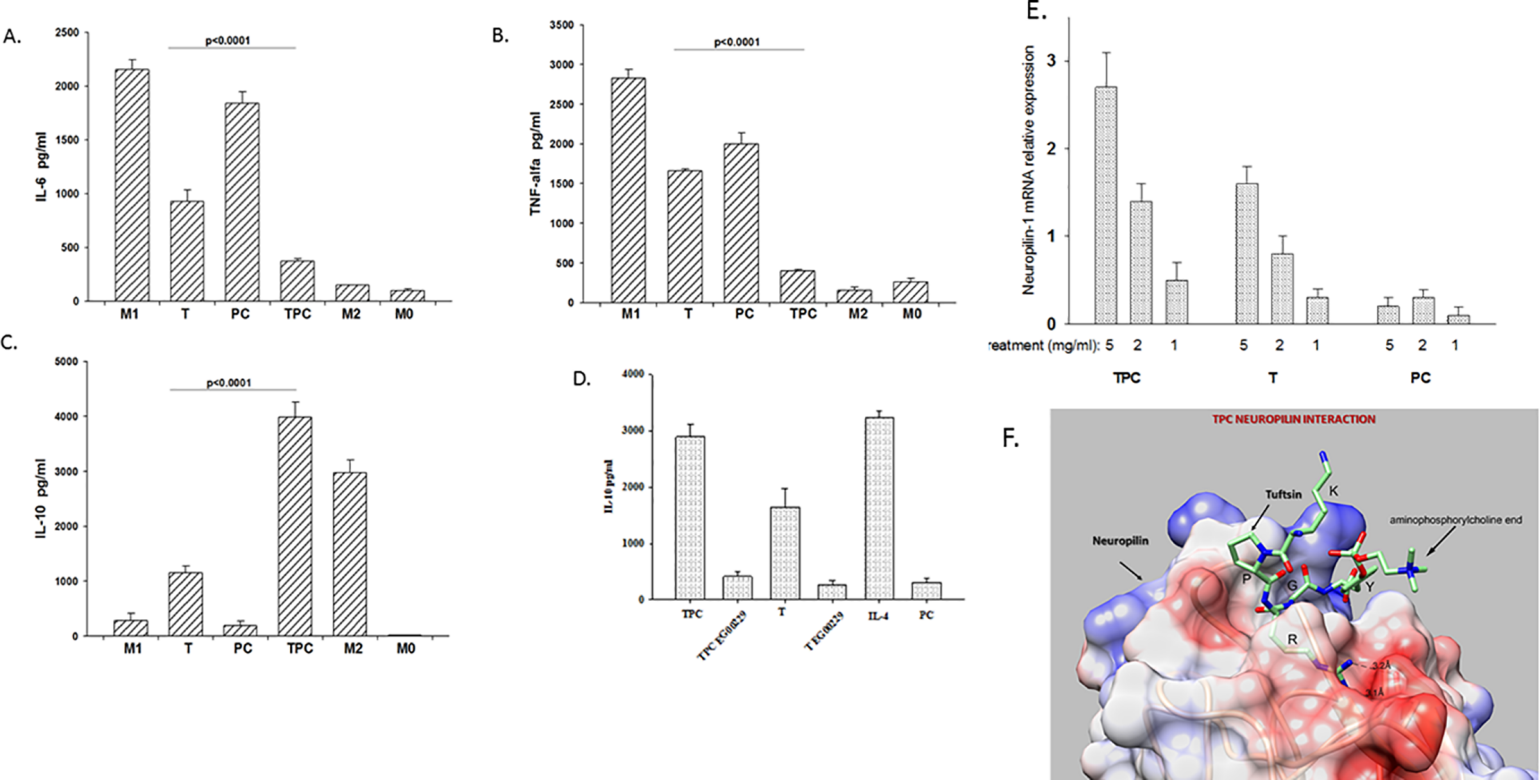
Cytokine expression in polarized RAW cells macrophages. In vitro analyses of **concentrations** of the pro-inflammatory cytokines IL-6, TNF-α, and the anti-inflammatory cytokine IL-10 in the culture fluids of M1 polarized RAW cells macrophages incubated with tuftsin-phosphoryl choline (TPC), tuftsin (T) and phosphorylcholine (PC). The data are presented as concentration in pg/ml. Values are the mean±SD, *P* < 0.001. [A] In vitro analyses of the pro-inflammatory cytokine IL-6 concentration. [B] In vitro analyses of the pro-inflammatory cytokine TNF-α concentration. [C] In vitro analyses of the anti-inflammatory cytokine IL-10 concentration. [D] TPC manipulate the shift from M1 toward M2 via neuropilin receptor. M1 RAW macrophages were incubated with neuropilin inhibitor EG00229, before adding TPC, T, and PC. The data are presented as mean ± SD of three repeated experiments (*P* < 0.001). [E] The effect of TPC on neuropilin mRNA expression in RAW cells by RT-PCR. The data are presented as mean ± SD of three repeated experiments. [F] Model structure of the TCP neuropilin complex. Tuftsin is shown as a stick diagram with carbon, nitrogen and oxygen atoms in green, blue and red, respectively. The surface of neuropilin is shown, colored by the electrostatic potential, blue for positive, red for negative and white for neutral. The surface was made transparent to show the binding of TPC residue R in a deep cavity and the bifurcated hydrogen bond between R and neuropilin residue D320.

Pro-inflammatory cytokines have a major impact on the recruitment of inflammatory agents to the inflamed area [1–10]. We evaluated *in-vitro* both splenocyte secretion of pro-inflammatory IL-1-β, IL-17, IL-6, TNF-α as well as anti-inflammatory IL-10 cytokines originating from the following groups of treated mice: TPC, PBS, tuftsin, and phosphorylcholine. The pro-inflammatory cytokine concentrations in TPC-treated mice were significantly lower compared to control PBS-, tuftsin-, and phosphorylcholine-treated mice (*P* < 0.001) (Fig 3A, Fig 3B, Fig 4C, Fig 4D, S1 File, S2 File, S6 File, S7 File).

The IL-1β level was decreased by 2.76 fold compared to PBS, by 2.8 compared to tuftsin, by 2.69 compared to phosphorylcholine, (*P* < 0.001 for all the compared groups). IL-17 level was diminished by a factor of 1.23 compared to PBS and by 1.25 compared to tuftsin (*P* < 0.001 for all the compared groups). IL-6 level was 1.75 times lower compared to PBS, 1.62 lower compared to tuftsin, 1.66 lower compared to phosphorylcholine, and 1.8 compared to untreated group (*P* < 0.001). The TNF-α level was reduced by 3.13 compared to PBS, by 3.15 compared to tuftsin, by 3.06 compared to phosphorylcholine, and by 3.08 compared to untreated (*P* < 0.001 for all the compared groups).

Moreover, TPC significantly increased secretion of anti-inflammatory cytokine IL-10 compared to CIA mice by 5.85 times, compared to PBS, by 5.74 compared to tuftsin, by 5.81 and compared to phosphorylcholine by 5.88 (*P* < 0.001 for all the compared groups) (Fig 3E, S4 File).

### TPC induces a shift of M1 inflammatory macrophages toward M2 anti-inflammatory macrophages *in-vitro*

To determine whether TPC, phosphorylcholine, or tuftsin can induce a shift from M1 to M2 polarized macrophages, M1 RAW 264.7 cells were induced by exposure to LPS and IFN-ɣ. M1 macrophages secreted high levels of IL-6 and TNF-α cytokines. TPC, phosphorylcholine, or tuftsin were applied to M1 macrophages. TPC caused a larger shift of M1 macrophages to M2 when compared to phosphorylcholine and tuftsin (*P* < 0.001) (Fig 4A, Fig 4B, Fig 4C, S3 File, S5 File, S8 File). For example, in the TPC-treated M1 cells, the pro-inflammatory cytokines IL-6 and TNF-α levels were significantly lower (mean of 373 pg/ml and 400 pg/ml, respectively), whereas the anti-inflammatory cytokine IL-10 level was significantly elevated (3982 pg/ml), leading to a shift of macrophages from M1 to M2. Yet, tuftsin-exposed M1 cells underwent less frequent switching to M2 cells (e.g., the IL-6 concentration reached 926 pg/ml, TNF-α 1658 pg/ml, and IL-10 1154 pg/ml). Incubation of M1 macrophages with phosphorylcholine did not cause a change to M2 (*P* < 0.05). Previously it was reported that tuftsin targets neuropilin-1 on microglia cells [26]. Likewise, as is demonstrated in Fig 4D, the shift to M2 by TPC can be attributed to neuropilin-1 receptor since the addition of EG002 neuropilin-1 inhibitor, decreased the secretion of IL-10 induced by TPC or tuftsin (*P* < 0.001), whereas phosphorylcholine did not have any effect on M2 phenotype shift.

Using RT-PCR, we have found that TPC enhanced the neuropilin-1 mRNA expression by RAW macrophage cells at stage of M2 (Fig 4E). Densitometry results depicted a significant neuropilin-1 expression when TPC was compared to phosphorylcholine (*P* < 0.02) or to tuftsin (*P* < 0.03). Phosphorylcholine had no effect on the neuropilin-1 mRNA level expressed by M2 cells.

### Model structure of the neuropilin-1/TPC complex

The most prominent interactions between the KPR peptide and neuropilin in the experimental X-ray structure (PDB code 2ORZ) are through the C-terminal R. Thus, R is located in a deep cavity of neuropilin-1, with its positive side chain forming a bifurcated hydrogen bond to neuropilin-1 residue D320. In our model of the complex of TPC with neuropilin-1 (Fig 4F), the TPC R side-chain maintained the bifurcated bond with neuropilin-1 D320. The C-terminus of TPC approached a positively charged surface region of neuropilin-1 (blue surface in Fig 4F) where it bound to the side chain of K351 and the backbone of K352. Notably, the structural changes required for accommodating TPC in the binding cavity of neuropilin-1 were minor, amounting to root mean square deviation (RMSD) of 0.35 Å for the Cα atoms of the whole chain. Our model shows that TPC can bind to neuropilin-1 and that the phosphorylcholine is exposed in the neuropilin-1/TPC complex, enabling it to bind to another molecule.

### TPC induced expansion of CD4^+^CD25^+^FOXP3^+^ Treg cells in CIA mice

**Fig 5:**
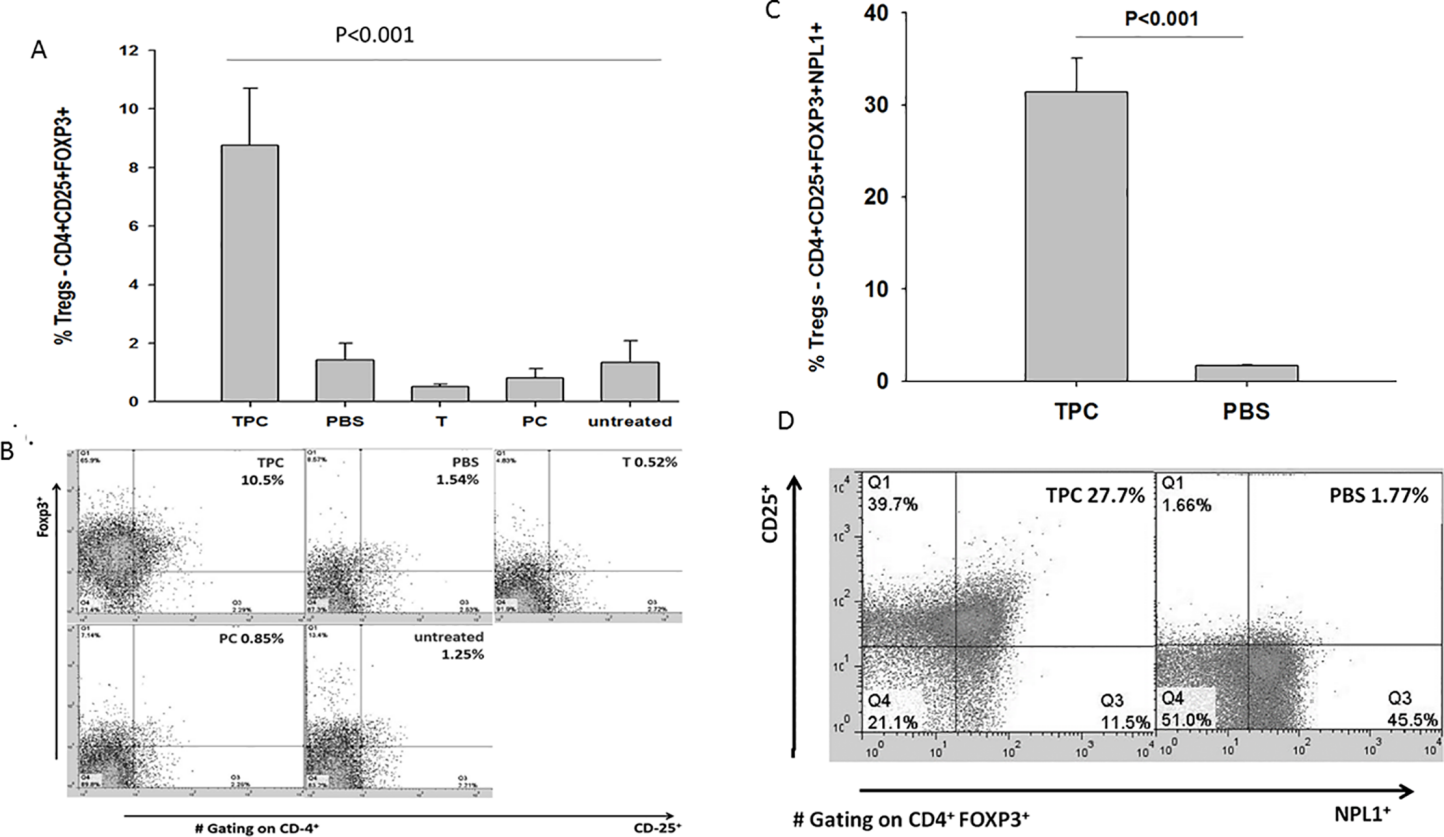
Increase in Treg cells in isolated splenocytes of TPC-treated CIA mice. [A] The data are presented as percentage of Treg cells -CD4^+^CD25^+^FOXP3^+^ expansion in isolated splenocytes of tuftsin- phosphorylcholine (TPC), PBS, tuftsin (T), phosphoryl choline (PC), and untreated CIA mice (n = 10). Values are the mean ± SD, *P* < 0.001. [B] Representative flow cytometry analyses of Treg cells -CD4^+^CD25^+^FOXP3^+^ (gated on CD4^+^) in isolated splenocytes derived from CIA mice treated with TPC, PBS, T and PC and untreated CIA mice. [C] The data are presented as percentage of Tregs -CD4^+^CD25^+^FOXP3^+^ neuropilin-1^+^ (NPN1^+)^ expansion in isolated splenocytes of TPC and PBS treated mice (n = 10). Values are the mean ± SD, *P* < 0.001. [D] Representative flow cytometry analyses of Tregs -CD4^+^CD25^+^FOXP3^+^ neuropilin-1^+^ NPN1^+^ (gated on CD4^+^FOXP3^+^) in splenocytes derived from the TPC and PBS treated mice *P* < 0.001.

TPC treatment significantly promoted the CD4^+^CD25^+^FOXP3^+^ Treg phenotype expansion when compared with control treated groups of mice (*P* < 0.001) (Fig 5A, Fig 5B). Splenocytes of TPC-treated CIA mice exhibited 8.75% Treg cells, whereas in the PBS group Treg level was 6.25 times lower than in the TPC treated mice (*P* < 0.001). When compared to TPC-subjected mice, the percentage of Treg in tuftsin-treated splenocytes was 16.8 times lower (*P* < 0.001), the phosphorylcholine Treg level was 10.8 times lower (*P* < 0.001), and the untreated CIA mice Treg level was 6.6 times lower (*P* < 0.001). Elevated numbers of Tregs positive for neuropilin-1 from total Tregs was defined by FACS analyses CD4^+^CD25^+^FOXP3^+^NPN1^+^ (gated on CD4^+^FOXP3^+^). Tregs neuropilin-1 (NRP1) positive splenocytes derived from the TPC were 27.7 ± 3% and compared to splenocytes from PBS treated mice 1.77 ± 0.4%, *P* < 0.001 (Fig 5C, Fig 5D).

### TPC induced expansion of IL-10^+^CD25^+^CD1d^+^ B10 regulatory cells in CIA mice

**Fig 6:**
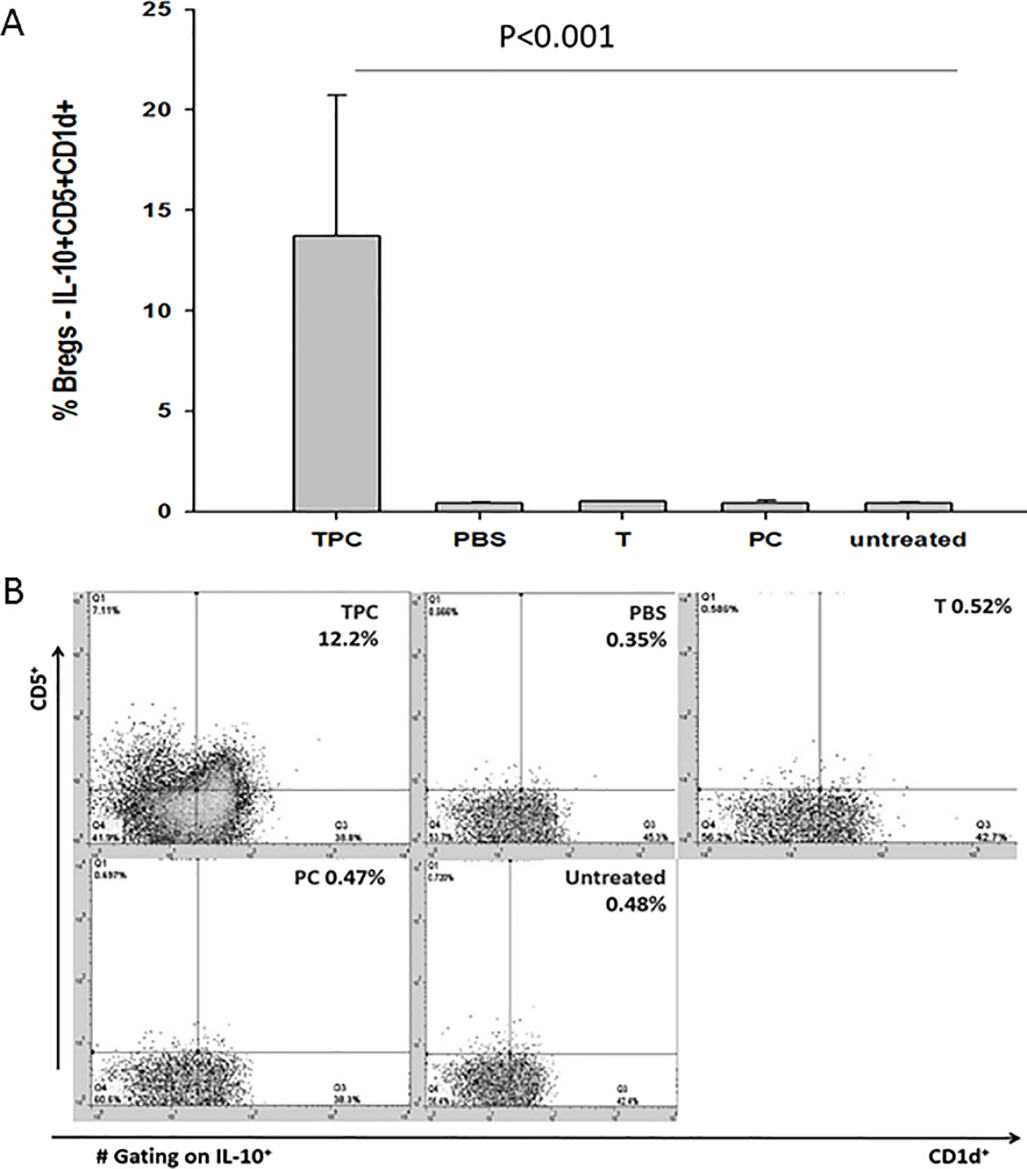
B10 regulatory cells increase in isolated splenocytes of TPC-treated CIA mice. [A] The data are presented as percentage of Breg cells–IL-10^+^CD5^+^CD1d^+^ increase in isolated splenocytes derived from tuftsin- phosphorylcholine (TPC), PBS, tuftsin (T), phosphorylcholine (PC), and untreated CIA mice (n = 10). Values are mean ± SD, *P* < 0.001. [B] Representative flow cytometry analyses of Breg cells–IL-10^+^CD5^+^CD1d^+^ (gated on IL-10^+^) in isolated splenocytes derived from CIA mice treated with TPC, PBS, T, and PC, as well as untreated CIA mice.

B cells were isolated by negative selection on Milteni Biotec magnetic beads, from isolated splenocytes derived from CIA mice, treated with TPC, PBS, tuftsin, and phosphorylcholine. The mean percentage of the IL-10^+^CD25^+^CD1d^+^ Breg cells subset in total B cells derived from the different groups of mice was determined (Fig 6A, Fig 6B). TPC significantly enhanced the amount of Breg cells expansion in isolated CIA mice splenocytes (*P* < 0.001) compared to CIA mice treated with PBS, tuftsin, and phosphorylcholine. The mean percentage of IL-10^+^CD25^+^CD1d^+^ Breg cells of TPC-subjected mice was 13.73%, while the PBS Breg level was 34 times lower (*P* < 0.001). Tuftsin level was 27.4 times lower (*P* < 0.001), phosphorylcholine Breg level was 31.2 times lower (*P* < 0.001), respectively.

### Phosphorylcholine site of TPC inhibited TLR4 expression by HEKbluemTLR4 cells *in-vitro*

**Fig 7:**
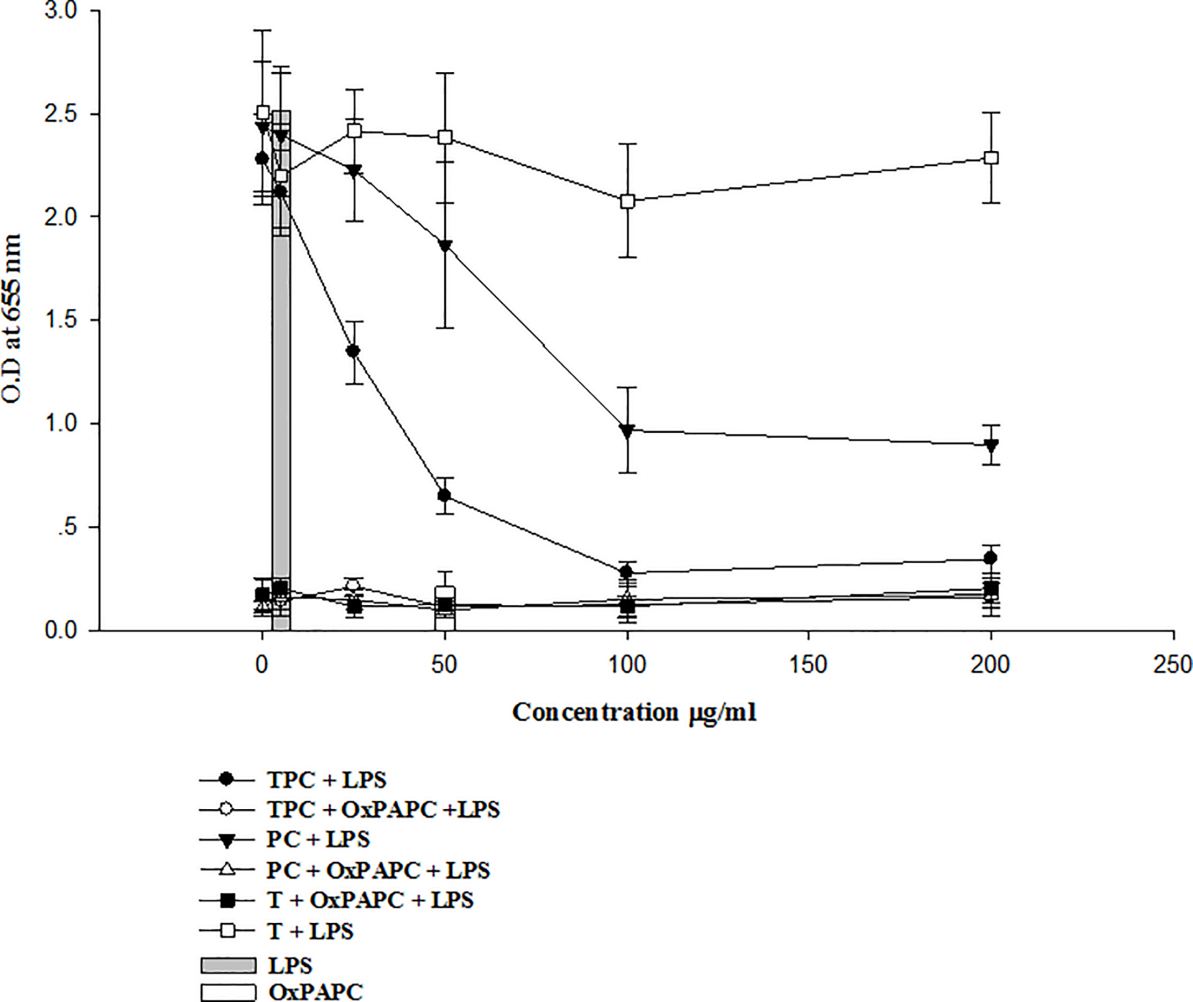
TPC or PC inhibits HEKblue mTLR4 expression. TPC inhibition of HEKblue mTLR4 activation by LPS, at different concentrations *via* detection of NF-kB expression. OxPAPC was used as TLR4 inhibitor. The data are presented as mean ± SD of three separate experiments.

To analyze whether TPC affects NF-kB expression *via* TLR4 pathway, we used the human embryonic kidney cells HEKblueTLR4 system. HEK cells were pre-incubated with TPC, phosphorylcholine, tuftsin +/- the OxPAPC TLR4 commercial blocker, and then stimulated with LPS. As illustrated in Fig 7, OxPAPC (50 μg/ml) abrogated NF-kB *via* TLR4 expression (*P* < 0.001) compared to LPS induced NF-kB expression (gray and white columns). TPC at the similar concentration reduced the TLR4-LPS activation by 74% (filled circles) (*P* < 0.001), compared to OxPAPC blockage. Phosphorylcholine inhibited LPS activation by 33% (filled triangles) at concentrations of 100 μg/ml. TPC damped the TLR4 expression by 84% and phosphorylcholine by 61%, respectively. Tuftsin had no effect on HEK TLR4 activation by LPS (empty square) (*P* > 0.05) when compared to LPS alone. Pre-incubation of HEK cells with OxPAPC before adding TPC or phosphorylcholine, diminished the TLR4 expression (Fig 7).

## Discussion

A decade ago the phosporylcholine moiety of the filarial nematode was found to be an immunomodulator in a host microenvioroment [21]. We conjugated phosphorylcholine to a self immunomodulatory molecule, tuftsin, and named this small molecule—TPC. Tuftsin, having the sequence Thr-Lys-Pro-Arg, naturally occurs in human blood. This peptide is a fragment of the heavy chain Fc (289–292) of an IgG. Tuftsin is an endogenous immunomodulator of a wide spectrum of biological activity, It enhances phagocytosis, chemotaxis and pinocytosis and has antimicrobial and anticoagulant properties. Tuftsin targets FcγR and neuropilin-1. It is used as an adjuvant for mucosal vaccine based on HE-ORF2 and HA-VP1 (Hepatitis E virus and Hepatitis A virus) [22, 31].

Our current study shows the capability of TPC to mitigate arthritis progression and inflammation condition in established murine CIA. Joints of mice treated with PBS, tuftsin, and phosphorylcholine, were highly inflamed, compared to TPC treated CIA mice. TPC treatment inhibited secretion of the key pro-inflammatory cytokines such as IL-1-β, IL-17, IL-6, and TNF-α and increased production of the anti-inflammatory cytokine IL-10, resulting in a lowered inflammatory state. An additional source of IL-10 may be related to TPC causing shift of macrophages from M1 to M2 secreting IL-10. Based on our *in-vitro* studies one may link the enhanced IL-10 levels triggered by TPC to the following sources: (a) enhanced expansion of T regulatory cells and B regulatory cells both secreting IL-10 and (b) shifting macrophages from inflammatory M1 to IL-10-secreting M2 macrophages. Phosphorylcholine alone did not cause a switch from M1 to M2 macrophages. The mode of TPC activity by which TPC binds and affects regulatory cells and macrophages involves neuropilin-1. The present data show that TPC binds the neuropilin-1 on macrophages and Treg cells causing enhanced secretion of IL-10. Commercial neuropilin-1 inhibitor, reduced the tuftsin component of TPC binding to macrophages and IL-10 secretion by the cells. Furthermore, TPC enhanced the expression of M2 macrophages neuropilin-1 mRNA upon exposure to TPC and to a lesser extent by incubation with tuftsin, as confirmed by RT-PCR. This effect correlates with tuftsin ability to induce an anti-inflammatory M2 phenotype *via* binding to neuropilin-1 in microglia cells [22,26,32,33]. Neuropilin-1 is a non-tyrosine kinase cell membrane surface glycoproteins, which serve as co-receptor for class III semaphorins, and for members of the vascular endothelial growth factor due to shared epitope amino acid sequence [34]. Neuropilins have a role in immune cell communications and immunomodulation of immune network [34]. Neuropilin-1 is expressed by different immune cells including, macrophages, dendritic cells leading to tolerance induction, and T and B cell subsets, especially regulatory T cell populations [34]. The cross talk between tuftsin and neuropilin-1 showed that tuftsin targeting neuropilin-1microglia and trigger the cells and promoting the M2 anti-inflammatory phenotype, and attenuate experimental autoimmune encephalomyelitis (EAE) [26].

Our data point to the expansion of Breg cells and Treg cells in CIA mice treated with TPC. Treg cells and Breg cells are immunosuppressive cells that support immunological tolerance [35–37]. Treg cells CD25+CD4+FOXP3+ are required for the maintenance of immune self-tolerance and homeostasis by suppressing aberrant or excessive immune responses which is harmful to the host. Expansion of Treg cells in autoimmunity is essential to maintain tolerance. TPC enlarge the number of peripheral Tregs in our CIA mice. The transcription factor FOXP3 plays a key role in Treg cell development and function suppressing various effector lymphocytes, especially helper T cell subsets: Th1, Th2, Th17 [35,36]. During recent years epigenetic contribution in concert with FOXP3 was shown to be crucial for Treg development and function [36]. One of the mechanisms of Treg-mediated suppression is the secretion of anti-inflammatory cytokines IL- 10 and TGF-β [35, 36].

There is strong evidence that the number of Breg cells and its suppressive activity, increase in response to inflammation during several autoimmune disorders such as experimental arthritis, experimental autoimmune encephalomyelitis [36–40]. Through the production of IL-10, TGFβ, IL-35, Bregs control the differentiation of lymphocytes secreting pro-inflammatory cytokines such as TNF-αproducing monocytes, IL12 producing dendritic cells, Th17 cells and differentiation of T regulatory cells [36]. Likewise Bregs can arise in response to autoimmune related proinflammatory cytokines IL-6, IL-1β that are produced in response to an antigen, such as in an athrthritis murine experimental model, preventing the uncontrolled expansion of pro-inflammatory lymphocytes such as Th17 cells. [41]. The cellular and molecular basis of TPC-mediated Treg and Breg cells expansion and function needs further investigations.

TPC and to a lesser extent phosphorylcholine, inhibited TLR4 expression using HEKblue-mouse TLR4 (mTLR4) system. TLR4 requires MD-2, a secreted molecule, to interact functionally with LPS. The CD14 protein, participated in LPS signaling, leading to NF-κB translocation. This signaling is mediated through several adaptor proteins, including MyD88 TIRAP/Mal, TRIF/TICAM1, and TRAM/TICAM2 [42]. The inhibitor 1-palmitoyl-2-arachidonyl-sn-glycero-3-phosphorylcholine (OxPAPC) is restricted to TLR4 and inhibits TLR4 expression by the HEK-blue-mTLR4 cells [43]. TPC and phosphorylcholine significantly ameliorated TLR4 function, as demonstrated by secreted embryonic alkaline phosphatase reporter gene (SEAP) expression, while tuftsin did not affect TLR4 expression. Previous studies showed the targeting of TLR4 by phosphorylcholin, affecting the Myd88 pathway and reducing the inflammatory process via silencing NFkB [44].

Hence, as illustrated in Fig 4F, we hypothesize that because TPC is composed of two molecules (tuftsin and phosphorylcholine) the bi-specific function encompasses the phosphorylcholine site, inhibiting the TLR4 and the tuftsin/neuropilin-1 interaction, shifting the macrophage phenotype toward M2 anti-inflammatory secreting IL-10 as well as induction of Tregs expansion. The bi-specific activity of TPC may materialize in only one type of cells, or due to interaction between two types of cells expressing neuropilipi-1 or on one cell and TLR4.

Treatment with TPC induced the expansion of CD4+CD25+FOXP3+ Treg and CD5+CD1d+ B10 regulatory cells in isolated splenocytes; thus, modulating the cytokine profile and decreasing the level of inflammation and synovial hyperplasia. Prominent Treg and Breg cells enabled the decrease in pro-inflammatory cytokines IL-1-β, IL-6, and TNF-α and increased the anti-inflammatory cytokine IL-10 in stimulated, isolated splenocytes taken from TPC treated mice.

## Conclusions

This study was designed to assess the effect of helminth-derived phosphorylcholine conjugated to the self immunomodulatory molecule tuftsin, TPC, treatment on an established CIA. We have succeeded to show that TPC inhibits the clinical score of disease and the inflammatory process in the joints, as illustrated by clinical arthritis scoring system and histological assessment. The process of TPC immunomodulatory activity was illustrated by its ability to inhibit the secretion of pro-inflammatory cytokines, such as TNF-α, IL-1-β, and IL-6, and to upregulate IL-10 expression. The IL-10 source was Treg and Breg expansion as well as a possible conversion of macrophages to anti-inflammatory M2 macrophages secreting IL-10 *via* neuropilin-1. The amelioration of CIA was attributed to the bi-functional activity of TPC, targeting TLR4 through the phosphorylcholine leading to NF-kB inhibition and targeting neuropilin-1 *via* the tuftsin end of the molecule, leading to macrophages shift towards M2 anti-inflammatory secreting IL-10. The bi-functional activity resulted the anti-inflammatory network scenario. The molecular basis for all these functions should be further analyzed.

The results reported in the presented work may lead to a new potential treatment of RA patients with TPC, a small immunomodulatory molecule.

## Supporting information

S1 File (XLS)

S2 File (XLS)

S3 File (XLS)

S4 File (XLS)

S5 File (XLS)

S6 File (XLS)

S7 File (XLS)

S8 File (XLS)

S9 File (XLSX)
